# Hydrazine-1,2-diium bis­(3-carb­oxy-4-hy­droxy­benzene­sulfonate) tetra­hydrate

**DOI:** 10.1107/S1600536811014231

**Published:** 2011-04-29

**Authors:** Devipriya Selvaraju, Ranjithkumar Venkatesh, Vairam Sundararajan

**Affiliations:** a Department of Chemistry, Government College of Technology, Coimbatore 641 013, India

## Abstract

Reaction of 5-sulfosalicylic acid with hydrazine hydrate at pH = 1 results in the formation of the title hydrated salt, 0.5N_2_H_6_^2+^·C_7_H_5_O_6_S^−^·2H_2_O. The hydrazinium dications lie on centres of inversion. They are located between 3-carb­oxy-4-hy­droxy­benzene­sulfonate anions, forming inter­molecular N—H⋯O hydrogen bonds with sulfonate ions and water mol­ecules  of crystallisation. Further intra- and inter­molecular O—H⋯O hydrogen bonds are observed in the crystal structure.

## Related literature

For general background on hydrogen bonding in proton-transfer compounds of 3-carb­oxy-4-hy­droxy­benzene­sulfonate anions with Lewis bases, see: Smith *et al.* (2004 ▶, 2005 ▶). For recent related structures containing the 3-carb­oxy-4-hy­droxy­benzene­sulfonate anion, see: Wang, Yang *et al.* (2008 ▶); Wang, Yao *et al.* (2008 ▶); Smith & Wermuth (2009 ▶); Hemamalini & Fun (2010 ▶); Yin *et al.* (2010 ▶). For related structures containing the N_2_H_6_^2+^ hydrazinium dication, see: Starosta & Leciejewicz (2008 ▶); Klapotke *et al.* (1996 ▶).
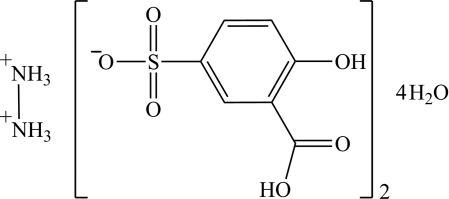


## Experimental

### 

#### Crystal data

0.5N_2_H_6_^2+^·C_7_H_5_O_6_S^−^·2H_2_O
*M* *_r_* = 270.25
Triclinic, 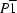

*a* = 7.0620 (5) Å
*b* = 7.2069 (4) Å
*c* = 11.5995 (8) Å
α = 78.460 (3)°
β = 75.806 (3)°
γ = 77.379 (3)°
*V* = 551.77 (6) Å^3^
*Z* = 2
Mo *K*α radiation
μ = 0.33 mm^−1^
*T* = 296 K
0.50 × 0.40 × 0.30 mm

#### Data collection

Bruker Kappa APEXII CCD area-detector diffractometer
Absorption correction: multi-scan (*SADABS*; Bruker, 1999 ▶) *T*_min_ = 0.823, *T*_max_ = 0.882
9320 measured reflections
2711 independent reflections
2582 reflections with *I* > 2σ(*I*)
*R*_int_ = 0.057

#### Refinement

*R*[*F* ^2^ > 2σ(*F* ^2^)] = 0.040
*wR*(*F* ^2^) = 0.107
*S* = 1.12
2711 reflections
187 parameters
6 restraints
H atoms treated by a mixture of independent and constrained refinement
Δρ_max_ = 0.41 e Å^−3^
Δρ_min_ = −0.52 e Å^−3^

### 

Data collection: *APEX2* (Bruker, 2004 ▶); cell refinement: *APEX2* and *SAINT* (Bruker, 2004 ▶); data reduction: *SAINT* and *XPREP* (Bruker, 2004 ▶); program(s) used to solve structure: *SIR92* (Altomare *et al.*, 1993 ▶); program(s) used to refine structure: *SHELXL97* (Sheldrick, 2008 ▶); molecular graphics: *ORTEP-3* (Farrugia, 1997 ▶) and *Mercury* (Bruno *et al.*, 2002 ▶); software used to prepare material for publication: *SHELXL97*.

## Supplementary Material

Crystal structure: contains datablocks I, global. DOI: 10.1107/S1600536811014231/zq2096sup1.cif

Structure factors: contains datablocks I. DOI: 10.1107/S1600536811014231/zq2096Isup2.hkl

Additional supplementary materials:  crystallographic information; 3D view; checkCIF report

## Figures and Tables

**Table 1:** Hydrogen-bond geometry (Å, °)

*D*—H⋯*A*	*D*—H	H⋯*A*	*D*⋯*A*	*D*—H⋯*A*
O6—H6⋯O5	0.82	1.88	2.6074 (18)	146
O7—H7*A*⋯O2^i^	0.83 (2)	1.98 (2)	2.8036 (15)	177 (2)
O7—H7*B*⋯O8	0.85 (2)	1.84 (2)	2.6754 (17)	171 (2)
O4—H4⋯O7^ii^	0.79 (3)	1.89 (3)	2.6758 (16)	174 (3)
O8—H8*B*⋯O2^iii^	0.86 (2)	2.00 (2)	2.8384 (16)	165 (2)
O8—H8*A*⋯O3^iv^	0.87 (2)	1.95 (2)	2.8213 (17)	177 (3)
N1—H1*A*⋯O3^v^	0.82 (2)	1.93 (2)	2.7493 (17)	175.2 (19)
N1—H1*B*⋯O7	0.94 (2)	1.84 (2)	2.7798 (16)	174.5 (19)
N1—H1*C*⋯O1^vi^	0.91 (2)	1.84 (2)	2.6813 (16)	154 (2)

Symmetry codes: (i) 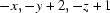; (ii) 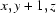; (iii) 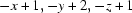; (iv) 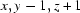; (v) 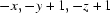; (vi) 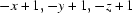.

**Fig. 1.**
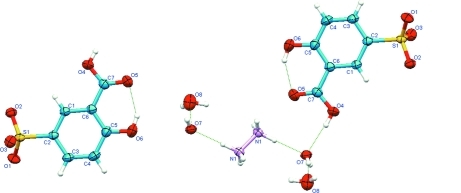
View of the title compound (I), showing displacement ellipsoids at the 50% probability level. Symmetry code: 1 - x, 1 - y, 1 - z.

**Fig. 2.**
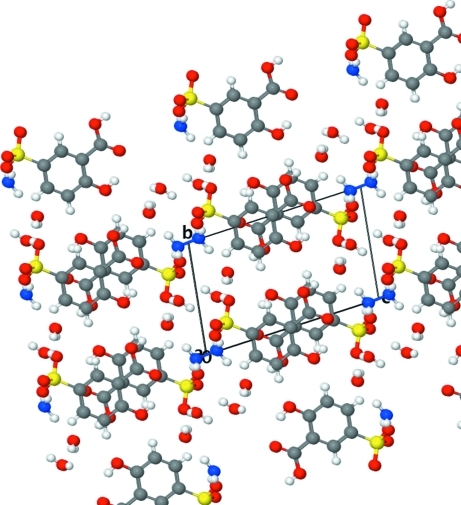
The packing diagram of title compound (I).

## supplementary crystallographic information

### Comment

5-Sulfosalicylic acid, a strong organic acid with pK_a_ = 2.85, donates its sulfonic protons to N-containing Lewis bases (Smith *et al.*, 2004, 2005) forming organic salts (Wang, Yang *et al.*, 2008; Wang, Yao *et al.*, 2008; Smith & Wermuth, 2009; Hemamalini & Fun, 2010; Yin *et al.*, 2010). Hydrazine as a diacidic base captures the H atoms of sulfonic groups from two acid molecules to form a dicationic hydrazinium salt. The molecular structure of the salt, C_7_H_5_O_6_S.0.5(H_6_N_2_).2(H_2_O), formed by the reaction of 5-sulfosalicylic acid with hydrazine hydrate at pH = 1 is shown in Fig.1 and the various types of hydrogen bonds involved in the crystal structure are reported in Table 1.

The unit cell of the crystal structure of the title compound contains eight (N_2_H_6_)^2+^ units located at the unit cell corners (centres of inversion) which contribute to 1/8 of the charge of each cell, two compensating 3-carboxy-4-hydroxybenzenesulfonate anions, and four isolated water molecules. The anions are held by intermolecular N—H···O hydrogen bonds in all directions with the hydrazinium ions. These N···O interactions fall in the range of 2.6813 (16)–2.7798 (16) Å. In addition, the intramolecular O6—H6···O5 hydrogen bond [*D*···*A* = 2.6074 (18) Å] and intermolecular O—H···O hydrogen bonds, O7—H7······O2 [*D*···*A* = 2.8036 (15) Å] between sulfonyl O atoms and water molecules, O4—H4···O7 [*D*···*A* = 2.6758 (16) Å] between carboxyl H atoms and isolated water molecules, and O7—H7B···O8 [*D*···*A* = 2.6754 (17) Å] between lattice water molecules, stabilize the molecular conformation and the crystal structure.

The N1—N1^i^ distance of 1.433 (2) Å (symmetry code: (i) -*x*, -*y*, -*z* + 2) is in a good agreement with the values reported by Starosta & Leciejewicz (2008) and Klapotke *et al.* (1996). The hydrazinium ion has a staggered conformation due to the symmetry imposed by the centre of inversion located in the middle of the N—N bond.

### Experimental

The title compound was synthesized by dissolving 5-sulfosalicylicacid dihydrate (2 mmol, 0.508 g) and hydrazine hydrate (99.98% pure; 1 mmol, 0.05 ml) in 30 ml of distilled water at pH = 1. The mixture was stirred for 4 h at ambient temperature and then filtered. The resulting clear solution was kept for three weeks in a wooden enclosure. Colourless prismatic crystals suitable for single-crystal X-ray diffraction analysis were grown by slow evaporation of the solvent.

### Refinement

All non-H atoms were refined anisotropically. H atoms bonded to C atoms were positioned geometrically with C—H = 0.93 Å and *U*_iso_(H) = 1.2*U*_eq_(C) while H atoms bonded to N and O atoms were found in difference Fourier maps and their coordinates and thermal parameters freely refined.

### Crystal data

**Table:**

0.5N_2_H_6_^2+^·C_7_H_5_O_6_S^−^·2H_2_O	*Z* = 2
*M**_r_* = 270.25	*F*(000) = 282
Triclinic, *P*1	*D*_x_ = 1.627 Mg m^−^^3^
Hall symbol: -P 1	Mo *K*α radiation, λ = 0.71073 Å
*a* = 7.0620 (5) Å	Cell parameters from 6221 reflections
*b* = 7.2069 (4) Å	θ = 2.5–26.2°
*c* = 11.5995 (8) Å	µ = 0.33 mm^−^^1^
α = 78.460 (3)°	*T* = 296 K
β = 75.806 (3)°	Block, colourless
γ = 77.379 (3)°	0.50 × 0.40 × 0.30 mm
*V* = 551.77 (6) Å^3^	

### Data collection

**Table:**

Bruker Kappa APEXII CCD area-detector diffractometer	2711 independent reflections
Radiation source: fine-focus sealed tube	2582 reflections with *I* > 2σ(*I*)
graphite	*R*_int_ = 0.057
ω and φ scans	θ_max_ = 28.2°, θ_min_ = 2.9°
Absorption correction: multi-scan (*SADABS*; Bruker, 1999)	*h* = −9→9
*T*_min_ = 0.823, *T*_max_ = 0.882	*k* = −9→9
9320 measured reflections	*l* = −13→15

### Refinement

**Table:**

Refinement on *F*^2^	Secondary atom site location: difference Fourier map
Least-squares matrix: full	Hydrogen site location: inferred from neighbouring sites
*R*[*F*^2^ > 2σ(*F*^2^)] = 0.040	H atoms treated by a mixture of independent and constrained refinement
*wR*(*F*^2^) = 0.107	*w* = 1/[σ^2^(*F*_o_^2^) + (0.0527*P*)^2^ + 0.1344*P*] where *P* = (*F*_o_^2^ + 2*F*_c_^2^)/3
*S* = 1.12	(Δ/σ)_max_ < 0.001
2711 reflections	Δρ_max_ = 0.41 e Å^−^^3^
187 parameters	Δρ_min_ = −0.52 e Å^−^^3^
6 restraints	Extinction correction: *SHELXL97* (Sheldrick, 2008), Fc^*^=kFc[1+0.001xFc^2^λ^3^/sin(2θ)]^-1/4^
Primary atom site location: structure-invariant direct methods	Extinction coefficient: 1.12 (4)

### Special details

**Table:**

Geometry. All e.s.d.'s (except the e.s.d. in the dihedral angle between two l.s. planes) are estimated using the full covariance matrix. The cell e.s.d.'s are taken into account individually in the estimation of e.s.d.'s in distances, angles and torsion angles; correlations between e.s.d.'s in cell parameters are only used when they are defined by crystal symmetry. An approximate (isotropic) treatment of cell e.s.d.'s is used for estimating e.s.d.'s involving l.s. planes.
Refinement. Refinement of *F*^2^ against ALL reflections. The weighted *R*-factor *wR* and goodness of fit *S* are based on *F*^2^, conventional *R*-factors *R* are based on *F*, with *F* set to zero for negative *F*^2^. The threshold expression of *F*^2^ > σ(*F*^2^) is used only for calculating *R*-factors(gt) *etc*. and is not relevant to the choice of reflections for refinement. *R*-factors based on *F*^2^ are statistically about twice as large as those based on *F*, and *R*-factors based on ALL data will be even larger.

### Fractional atomic coordinates and isotropic or equivalent isotropic displacement parameters (Å^2^)

**Table:**

	*x*	*y*	*z*	*U*_iso_*/*U*_eq_	
C1	0.28715 (19)	1.12543 (18)	0.37831 (12)	0.0277 (3)	
H7	0.2576	1.2577	0.3783	0.033*	
C2	0.36576 (19)	1.05458 (18)	0.27144 (12)	0.0268 (3)	
C3	0.4096 (2)	0.8553 (2)	0.27042 (15)	0.0373 (3)	
H1	0.4618	0.8079	0.1981	0.045*	
C4	0.3755 (3)	0.7304 (2)	0.37640 (16)	0.0434 (4)	
H3	0.4049	0.5983	0.3756	0.052*	
C5	0.2966 (2)	0.8008 (2)	0.48587 (14)	0.0364 (3)	
C6	0.2515 (2)	1.00021 (19)	0.48664 (13)	0.0290 (3)	
C7	0.1686 (2)	1.0757 (2)	0.60113 (13)	0.0323 (3)	
N1	0.01665 (18)	−0.00608 (18)	0.93729 (10)	0.0272 (3)	
O1	0.59710 (15)	1.13796 (16)	0.06468 (10)	0.0369 (3)	
O7	−0.03247 (17)	0.36335 (14)	0.80737 (10)	0.0340 (3)	
O2	0.40796 (15)	1.40062 (14)	0.17028 (9)	0.0333 (3)	
O8	0.19337 (19)	0.5388 (2)	0.88950 (13)	0.0505 (3)	
O3	0.24335 (15)	1.22941 (15)	0.07799 (10)	0.0356 (3)	
O4	0.1284 (2)	1.26416 (17)	0.58929 (11)	0.0445 (3)	
O5	0.1392 (2)	0.97087 (18)	0.69881 (10)	0.0454 (3)	
O6	0.2667 (2)	0.67267 (18)	0.58729 (12)	0.0568 (4)	
H6	0.2187	0.7301	0.6450	0.085*	
S1	0.40819 (4)	1.21604 (4)	0.13623 (3)	0.02543 (16)	
H7A	−0.144 (2)	0.430 (3)	0.8122 (19)	0.046 (5)*	
H7B	0.043 (3)	0.425 (3)	0.825 (2)	0.062 (7)*	
H4	0.086 (4)	1.300 (4)	0.653 (2)	0.058 (7)*	
H8B	0.312 (3)	0.559 (4)	0.858 (2)	0.069 (7)*	
H8A	0.208 (4)	0.446 (3)	0.949 (2)	0.091 (10)*	
H1A	−0.060 (3)	−0.070 (3)	0.9285 (17)	0.035 (5)*	
H1B	−0.008 (3)	0.118 (3)	0.8930 (19)	0.045 (5)*	
H1C	0.144 (3)	−0.067 (3)	0.9181 (19)	0.047 (5)*	

### Atomic displacement parameters (Å^2^)

**Table:**

	*U*^11^	*U*^22^	*U*^33^	*U*^12^	*U*^13^	*U*^23^
C1	0.0271 (6)	0.0250 (6)	0.0312 (6)	−0.0017 (5)	−0.0065 (5)	−0.0078 (5)
C2	0.0240 (6)	0.0262 (6)	0.0300 (6)	−0.0008 (4)	−0.0065 (5)	−0.0067 (5)
C3	0.0428 (8)	0.0286 (7)	0.0387 (8)	0.0024 (6)	−0.0068 (6)	−0.0130 (6)
C4	0.0567 (10)	0.0237 (6)	0.0467 (9)	0.0024 (6)	−0.0108 (8)	−0.0092 (6)
C5	0.0438 (8)	0.0273 (7)	0.0374 (7)	−0.0021 (6)	−0.0134 (6)	−0.0018 (6)
C6	0.0289 (6)	0.0291 (6)	0.0303 (6)	−0.0027 (5)	−0.0090 (5)	−0.0069 (5)
C7	0.0331 (7)	0.0350 (7)	0.0300 (7)	−0.0061 (5)	−0.0079 (5)	−0.0063 (5)
N1	0.0263 (6)	0.0313 (6)	0.0243 (6)	−0.0042 (4)	−0.0055 (4)	−0.0061 (4)
O1	0.0251 (5)	0.0461 (6)	0.0361 (5)	−0.0001 (4)	0.0002 (4)	−0.0140 (5)
O7	0.0362 (6)	0.0295 (5)	0.0361 (5)	−0.0013 (4)	−0.0069 (4)	−0.0105 (4)
O2	0.0336 (5)	0.0292 (5)	0.0376 (5)	−0.0074 (4)	−0.0033 (4)	−0.0094 (4)
O8	0.0391 (7)	0.0573 (8)	0.0577 (8)	−0.0184 (6)	−0.0190 (6)	0.0066 (6)
O3	0.0306 (5)	0.0376 (5)	0.0417 (6)	−0.0068 (4)	−0.0152 (4)	−0.0031 (4)
O4	0.0646 (8)	0.0348 (6)	0.0307 (6)	−0.0039 (5)	−0.0029 (5)	−0.0115 (4)
O5	0.0622 (8)	0.0433 (6)	0.0285 (5)	−0.0086 (5)	−0.0079 (5)	−0.0034 (5)
O6	0.0906 (11)	0.0314 (6)	0.0418 (7)	−0.0058 (6)	−0.0132 (7)	0.0033 (5)
S1	0.0201 (2)	0.0276 (2)	0.0285 (2)	−0.00186 (12)	−0.00404 (13)	−0.00819 (13)

### Geometric parameters (Å, °)

**Table:**

C1—C2	1.3775 (19)	N1—N1^i^	1.433 (2)
C1—C6	1.3954 (19)	N1—H1A	0.82 (2)
C1—H7	0.9300	N1—H1B	0.94 (2)
C2—C3	1.4030 (18)	N1—H1C	0.91 (2)
C2—S1	1.7594 (14)	O1—S1	1.4495 (10)
C3—C4	1.374 (2)	O7—H7A	0.826 (15)
C3—H1	0.9300	O7—H7B	0.847 (15)
C4—C5	1.403 (2)	O2—S1	1.4610 (10)
C4—H3	0.9300	O8—H8B	0.859 (16)
C5—O6	1.3453 (19)	O8—H8A	0.874 (17)
C5—C6	1.404 (2)	O3—S1	1.4599 (10)
C6—C7	1.473 (2)	O4—H4	0.79 (3)
C7—O5	1.2282 (18)	O6—H6	0.8200
C7—O4	1.3115 (19)		
			
C2—C1—C6	120.48 (12)	O5—C7—C6	122.77 (14)
C2—C1—H7	119.8	O4—C7—C6	114.08 (13)
C6—C1—H7	119.8	N1^i^—N1—H1A	108.5 (13)
C1—C2—C3	120.22 (13)	N1^i^—N1—H1B	109.8 (13)
C1—C2—S1	119.44 (10)	H1A—N1—H1B	109.2 (19)
C3—C2—S1	120.34 (11)	N1^i^—N1—H1C	104.8 (14)
C4—C3—C2	119.94 (14)	H1A—N1—H1C	110.6 (18)
C4—C3—H1	120.0	H1B—N1—H1C	113.7 (18)
C2—C3—H1	120.0	H7A—O7—H7B	108.4 (18)
C3—C4—C5	120.36 (13)	H8B—O8—H8A	104 (2)
C3—C4—H3	119.8	C7—O4—H4	111.4 (18)
C5—C4—H3	119.8	C5—O6—H6	109.5
O6—C5—C4	118.15 (13)	O1—S1—O3	112.19 (7)
O6—C5—C6	122.23 (14)	O1—S1—O2	112.97 (6)
C4—C5—C6	119.63 (14)	O3—S1—O2	110.87 (6)
C1—C6—C5	119.37 (13)	O1—S1—C2	107.30 (6)
C1—C6—C7	120.56 (12)	O3—S1—C2	106.83 (6)
C5—C6—C7	120.07 (13)	O2—S1—C2	106.24 (6)
O5—C7—O4	123.15 (14)		
			
C6—C1—C2—C3	0.4 (2)	C4—C5—C6—C7	180.00 (15)
C6—C1—C2—S1	179.78 (10)	C1—C6—C7—O5	−178.27 (14)
C1—C2—C3—C4	−0.5 (2)	C5—C6—C7—O5	1.3 (2)
S1—C2—C3—C4	−179.82 (13)	C1—C6—C7—O4	2.0 (2)
C2—C3—C4—C5	0.1 (3)	C5—C6—C7—O4	−178.35 (14)
C3—C4—C5—O6	−179.70 (16)	C1—C2—S1—O1	139.04 (11)
C3—C4—C5—C6	0.3 (3)	C3—C2—S1—O1	−41.61 (14)
C2—C1—C6—C5	0.0 (2)	C1—C2—S1—O3	−100.46 (12)
C2—C1—C6—C7	179.62 (12)	C3—C2—S1—O3	78.89 (13)
O6—C5—C6—C1	179.65 (14)	C1—C2—S1—O2	17.95 (13)
C4—C5—C6—C1	−0.4 (2)	C3—C2—S1—O2	−162.70 (12)
O6—C5—C6—C7	0.0 (2)		

Symmetry codes: (i) −*x*, −*y*, −*z*+2.

### Hydrogen-bond geometry (Å, °)

**Table:**

*D*—H···*A*	*D*—H	H···*A*	*D*···*A*	*D*—H···*A*
O6—H6···O5	0.82	1.88	2.6074 (18)	146.
O7—H7A···O2^ii^	0.83 (2)	1.98 (2)	2.8036 (15)	177 (2)
O7—H7B···O8	0.85 (2)	1.84 (2)	2.6754 (17)	171 (2)
O4—H4···O7^iii^	0.79 (3)	1.89 (3)	2.6758 (16)	174 (3)
O8—H8B···O2^iv^	0.86 (2)	2.00 (2)	2.8384 (16)	165 (2)
O8—H8A···O3^v^	0.87 (2)	1.95 (2)	2.8213 (17)	177 (3)
N1—H1A···O3^vi^	0.82 (2)	1.93 (2)	2.7493 (17)	175.2 (19)
N1—H1B···O7	0.94 (2)	1.84 (2)	2.7798 (16)	174.5 (19)
N1—H1C···O1^vii^	0.91 (2)	1.84 (2)	2.6813 (16)	154 (2)

Symmetry codes: (ii) −*x*, −*y*+2, −*z*+1; (iii) *x*, *y*+1, *z*; (iv) −*x*+1, −*y*+2, −*z*+1; (v) *x*, *y*−1, *z*+1; (vi) −*x*, −*y*+1, −*z*+1; (vii) −*x*+1, −*y*+1, −*z*+1.
